# Comparative Transcriptome and iTRAQ Proteome Analyses of Citrus Root Responses to *Candidatus* Liberibacter asiaticus Infection

**DOI:** 10.1371/journal.pone.0126973

**Published:** 2015-06-05

**Authors:** Yun Zhong, Chun-zhen Cheng, Nong-hui Jiang, Bo Jiang, Yong-yan Zhang, Bo Wu, Min-lun Hu, Ji-wu Zeng, Hua-xue Yan, Gan-jun Yi, Guang-yan Zhong

**Affiliations:** 1 Institute of Fruit Tree Research, Guangdong Academy of Agricultural Sciences, Guangzhou, 510640, P.R.China; 2 Key Laboratory of South Subtropical Fruit Biology and Genetic Resource Utilization, Ministry of Agriculture, Guangzhou, 510640, P.R.China; 3 Key Laboratory of Tropical and Subtropical Fruit Tree Researches, Guangdong Province, Guangzhou, 510640, P.R.China; Key Laboratory of Horticultural Plant Biology (MOE), CHINA

## Abstract

Root samples of ‘Sanhu’ red tangerine trees infected with and without *Candidatus* Liberibacter asiaticus (*C*Las) were collected at 50 days post inoculation and subjected to RNA-sequencing and isobaric tags for relative and absolute quantification (iTRAQ) to profile the differentially expressed genes (DEGs) and proteins (DEPs), respectively. Quantitative real-time PCR was subsequently used to confirm the expression of 16 selected DEGs. Results showed that a total of 3956 genes and 78 proteins were differentially regulated by HLB-infection. Among the most highly up-regulated DEPs were sperm specific protein 411, copper ion binding protein, germin-like proteins, subtilisin-like proteins and serine carboxypeptidase-like 40 proteins whose transcript levels were concomitantly up-regulated as shown by RNA-seq data. Comparison between our results and those of the previously reported showed that known HLB-modulated biological pathways including cell-wall modification, protease-involved protein degradation, carbohydrate metabolism, hormone synthesis and signaling, transcription activities, and stress responses were similarly regulated by HLB infection but different or root-specific changes did exist. The root unique changes included the down-regulation in genes of ubiquitin-dependent protein degradation pathway, secondary metabolism, cytochrome P450s, UDP-glucosyl transferases and pentatricopeptide repeat containing proteins. Notably, nutrient absorption was impaired by HLB-infection as the expression of the genes involved in Fe, Zn, N and P adsorption and transportation were significantly changed. HLB-infection induced some cellular defense responses but simultaneously reduced the biosynthesis of the three major classes of secondary metabolites, many of which are known to have anti-pathogen activities. Genes involved in callose deposition were up-regulated whereas those involved in callose degradation were also up-regulated, indicating that the sieve tube elements in roots were hanging on the balance of life and death at this stage. In addition, signs of carbohydrate starvation were already eminent in roots at this stage. Other interesting genes and pathways that were changed by HLB-infection were also discussed based on our findings.

## Introduction

Citrus Huanglongbing (HLB), the most destructive and still uncontrollable disease, has been responsible for the loss of tens of millions of trees worldwide and generated tremendous economic losses to the citrus industries [1–3]. The causative agents, *Candidatus* Liberibacters, are gram-negative, phloem-inhabiting *α-Proteobacteria*. Currently three species, i.e., the heat-tolerant *Ca*. Liberibacter asiaticus (*C*Las), the heat-sensitive *Ca*. L. africanus (*C*Laf) and the recently identified heat tolerant *Ca*. L. americanus (*C*Lam), have been identified on *Citrus* [3, 4]. It is known that *C*Las is the most pathogenic of the three species. *C*Las can be transmitted by grafting with HLB-infected budwoods and by phloem-feeding psyllid, *Diaphorina citri*. Traditionally, HLB management practices include use of disease free nursery trees, control of citrus psyllid, and eradication of infected trees [3, 5], but the effectiveness of these measures varies with the levels of social and technical development. Efforts in breeding for HLB-resistance have not yielded satisfactory results for lack of resistance genes in *Citrus* [3]. Pyramiding resistance genes may offer a possible solution to HLB as shown by breeding for disease resistance in rice, wheat and barley [6–8]. But this strategy requires a through understanding of the host-pathogen interactions especially the QTLs involved.

Following colonization in citrus trees, the HLB pathogen disperses quickly and will spread to all tissues and organs including roots within three months to one year. Typical symptoms on diseased trees include variegated chlorosis of leaves, abnormal coloration of fruit, and dieback of twigs, among others, and eventually collapse of the trees in 2 to 5 years [3]. Fruit quality is adversely affected and often sourer and bitter [9]. Seed germination and seedling growth are also affected [10]. Biochemically, infected trees accumulate unusual high levels of starches in photosynthetic cells, phloem elements and vascular parenchyma cells of leaves and petioles, and in cells of xylem parenchyma and phelloderm of stems [11, 12]. The accumulation of starches is assumed to be resulted from phloem plugging associated with not only the bacteria growing inside but also callose deposition and accumulation of phloem proteins [13]. Chlorosis of leaves and shoots was reported to have a link with deficiency in minerals of N, Fe, Mg, and Zn [14, 15], and with disruption of chloroplast inner grana structure caused by excessively accumulated starches [13]. Transcriptomic studies showed that photosynthesis and carbohydrate metabolism genes, among many others, are abnormally expressed in leaves [16–22], stems [9] and fruits [23, 24]. In general, starch metabolism genes are down-regulated in all examined tissues, and photosynthesis genes are down-regulated in leaves and stems but not in fruits [23, 24]. MicroRNA (miRNA) profiles were reportedly changed in leaves and the findings that the phosphorus-starvation induced miR399 was significantly induced and that the P level was significantly reduced had inspired the authors to apply phosphorus to remit the diseased trees, which was reportedly to have significantly alleviated HLB symptoms [25].

It has long been assumed that the roots of HLB-infected trees should have suffered from starvation of carbohydrates [3, 26]. This is because the phloem cells constituting the carbohydrate passage from leaves to roots will be gradually blocked directly or indirectly by the continuously multiplying bacteria, resulting in an increasing reduction in the supply of carbohydrates to roots. Apparently, carbohydrate starvation will reduce the growth and activity of the roots, which will, in turn, cause a reduction in absorption and supply of minerals to the above ground tissues, thus further aggravating chlorosis symptoms. Indeed, starch depletion was observed in diseased roots by microscopy studies [9]. A more than 30% of reduction in fibrous root mass was observed in *C*Las infected sweet orange (*Citrus sinensis*) [27]. Changes in expression of 111 genes were also found in *C*Las infected roots [9]. Even though, evidence accumulated so far is not sufficient to elucidate the role of roots in the development of HLB symptoms and the molecular events induced by HLB-infection.

Global changes in expression of genes have provided valuable clues to the elucidation of the pathogenesis of plant diseases including bacterial diseases [3]. Modern genetics studies have shown that most genes exercise their roles through their protein products. Intuitively, a gene’s high level of transcripts should represent a correspondingly high level of its proteins. However, it has been extensively documented that posttranscriptional regulations, including translation, post-translational modification and degradation of the proteins determine to a large extent the actual levels of almost all proteins [28]. Therefore, to obtain a more correct picture about the role of a gene, its protein level should also be measured. In this study, we used respectively the RNA-seq and an eight-channel iTRAQ (isobaric tags for relative and absolute quantification) technique to analyze the genome-wide gene expression changes and to identify differentially expressed proteins in roots of ‘Sanhu’ red tangerine (*C*. *reticulata* Blanco cv. ‘Sanhu’) infected with *C*Las bacteria. ‘Sanhu’ red tangerine is a traditional rootstock and widely used in south China for the production of two important mandarins, ‘Shatangju’ (*C*. *reticulata* Blanco cv. ‘Shatangju’) and ‘Gonggan’ (*C*. *reticulata* Blanco cv. ‘Gonggan’). However, trees on ‘Sanhu’ red tangerine are very susceptible to *C*Las. Our aim is to identify the early *C*Las-responsive genes in roots with the hope that some of them may be exploited in breeding for HLB resistance and in developing a method to mitigating HLB symptoms of the diseased trees in the future.

## Results

### Comparative transcriptome analysis of *C*Las-affected and control ‘Sanhu’ red tangerine roots by Illumina sequencing

Two-year-old ‘Sanhu’ red tangerine seedlings were treated by graft-inoculation of *C*Las-carrying buds and healthy buds respectively. The inoculated trees were detected by PCR for the presence of *C*Las bacteria in leaves and roots once every 10 days. It was shown that no inoculated trees became HLB positive in both leaves and roots until 50 dpi (days post inoculation). These early HLB positive trees were then sampled together with the control trees for roots, from which total RNA and total protein were extracted.

RNA-seq generated approximately 52.9 million and 49.6 million 90-bp pair-end reads respectively for HLB-free control (C) and HLB-infected (H) root RNA samples. About 79.72% of C and 78.53% of H total reads were successfully mapped to *C*. *clementina* genome, and 63.06% and 60.72% of them matched with their corresponding transcripts in the archived gene sequence databases.

A ∣log_2_[fold change]∣ of ≥ 1 (p-value <0.005, FDR ≤0.001) was used to identify differentially expressed genes (DEGs). As a result, a total of 3956 DEGs were identified, 1840 of them were up-regulated and the rest 2116 were down-regulated (S1 Table). Kyoto encyclopedia of genes and genomes (KEGG) analysis showed that 19 pathways were significantly enriched (p-value <0.05 and q-value <0.05) (Table 1). Notably, DEGs involved in ‘plant-pathogen interaction pathway’ accounted for about 19% of the DEGs assigned to different pathways (Table 1).

**Table 1:** The 19 significantly enriched pathways identified by Kyoto Encyclopedia of Genes and Genomes (KEGG) analysis (p-value <0.05; q-value <0.05).

Rank	Pathway	DEGs with pathway annotation (2312)	All genes with pathway annotation (14097)	p-value	q-value	Pathway ID
1	Stilbenoid, diarylheptanoid and gingerol biosynthesis	114 (4.93%)	360 (2.55%)	3.21E-13	3.85E-11	ko00945
2	Phenylpropanoid biosynthesis	134 (5.8%)	476 (3.38%)	3.92E-11	2.35E-09	ko00940
3	Plant-pathogen interaction	434 (18.77%)	2052 (14.56%)	5.92E-10	2.37E-08	ko04626
4	Limonene and pinene degradation	76 (3.29%)	253 (1.79%)	3.73E-08	1.12E-06	ko00903
5	Plant hormone signal transduction	255 (11.03%)	1170 (8.3%)	3.01E-07	7.21E-06	ko04075
6	Biosynthesis of secondary metabolites	394 (17.04%)	1966 (13.95%)	2.48E-06	4.96E-05	ko01110
7	Glucosinolate biosynthesis	25 (1.08%)	65 (0.46%)	1.59E-05	0.000273	ko00966
8	Flavonoid biosynthesis	98 (4.24%)	413 (2.93%)	6.26E-05	0.000938	ko00941
9	Tryptophan metabolism	33 (1.43%)	110 (0.78%)	0.000263	0.003487	ko00380
10	ABC transporters	34 (1.47%)	115 (0.82%)	0.000291	0.003487	ko02010
11	Phenylalanine metabolism	46 (1.99%)	171 (1.21%)	0.000322	0.003515	ko00360
12	alpha-Linolenic acid metabolism	37 (1.6%)	141 (1%)	0.001911	0.019111	ko00592
13	Zeatin biosynthesis	36 (1.56%)	138 (0.98%)	0.002433	0.02246	ko00908
14	Flavone and flavonol biosynthesis	48 (2.08%)	211 (1.5%)	0.009841	0.084352	ko00944
15	Metabolic pathways	540 (23.36%)	3066 (21.75%)	0.022176	0.177407	ko01100
16	Biosynthesis of unsaturated fatty acids	18 (0.78%)	70 (0.5%)	0.030787	0.230902	ko01040
17	Pentose and glucuronate interconversions	38 (1.64%)	173 (1.23%)	0.03315	0.233999	ko00040
18	Alanine, aspartate and glutamate metabolism	19 (0.82%)	77 (0.55%)	0.040075	0.267169	ko00250
19	Starch and sucrose metabolism	68 (2.94%)	341 (2.42%)	0.045885	0.289797	ko00500

### iTRAQ-based comparative proteomic analysis of *C*Las-affected and healthy ‘Sanhu’ red tangerine roots

A total of 1455 proteins were identified from *C*Las infected and control root samples. A one by one search found that 1233 (92.78%) of the 1455 identified proteins had corresponding transcripts in our RNA-seq data. In the end, 78 proteins were identified as differentially expressed proteins (DEPs) at p-value <0.05 and a cutoff value of >|±1.5|-fold, which included 46 up-regulated and 32 down-regulated proteins (Table 2 and S2 Table). An expression correlation analysis was performed between these proteins and their corresponding transcripts, and a Pearson correlation coefficient of ~0.41 was obtained, indicating a moderate positive correlation existed between transcriptomic and proteomic data (S1 Fig).

**Table 2:** Differentially expressed proteins identified in *C*Las-infected ‘Sanhu’ red tangerine roots.

Gene ID*	Gene description	Protein Fold change	Gene Fold change
clementine0.9_003979m/Ciclev10018836m	Sperm specific protein 411 (SSP411)	6.475	-1.523
clementine0.9_029368m/Ciclev10024168m	Copper ion binding protein	4.783	5.856
clementine0.9_028487m/Ciclev10030149m	Germin-like protein	4.137	4.720
clementine0.9_035803m/Ciclev10023551m	Subtilisin-like protease	3.976	4.047
clementine0.9_002904m/Ciclev10027863m	Subtilisin-like protease-like	3.926	5.530
clementine0.9_033258m/Ciclev10018003m	Germin-like protein	3.284	3.410
clementine0.9_030770m/Ciclev10018062m	SCPL40 (serine carboxypeptidase-like 40)	3.138	8.143
clementine0.9_028660m/Ciclev10007081m	Major allergen mal d1	2.989	1.762
clementine0.9_021650m/Ciclev10022211m	Trypsin and protease inhibitor family protein	2.964	-1.190
clementine0.9_003047m/Ciclev10000364m	Subtilisin-like protease	2.721	0.772
clementine0.9_021318m/Ciclev10029251m	GLP5 (germin-like protein 5)	2.169	0.552
clementine0.9_023327m/Ciclev10026538m	Lipid-associated family protein	2.139	-0.266
clementine0.9_026109m/Ciclev10029627m	40s ribosomal protein s25-2	2.095	1.206
clementine0.9_027200m/Ciclev10013189m	Ubiquinol-cytochrome c reductase complex 8.0 kDa protein	2.014	-
clementine0.9_017579m/Ciclev10028975m	Plasma membrane intrinsic protein 2;4	1.96	-1.694
clementine0.9_017092m/Ciclev10021381m	Pre-mRNA-splicing factor SF2	1.918	-0.912
clementine0.9_017463m/Ciclev10028959m	PR3 (basic chitinase)	1.905	-1.199
clementine0.9_024393m/Ciclev10022655m	Major pollen allergen Car b 1 isoforms 1A and 1B	1.881	0.265
clementine0.9_015410m/Ciclev10026050m	Peroxidase	1.859	0.592
clementine0.9_025955m/Ciclev10006259m	60s ribosomal protein L30	1.83	-
clementine0.9_024868m/Ciclev10029528m	PR4 (Pathogenesis-related 4)	1.788	1.230
clementine0.9_001766m/Ciclev10030651m	ATP binding protein	1.768	-0.641
clementine0.9_014340m/Ciclev10008748m	Cysteine-type peptidase	1.746	0.670
clementine0.9_024894m/Ciclev10029532m	40S ribosomal protein S23	1.739	0.611
clementine0.9_011012m/Ciclev10004931m	PKT3 (Peroxisomal 3-ketoacyl-CoA thiolase 3)	1.733	-0.449
clementine0.9_021972m/Ciclev10006241m	23.5 kDa mitochondrial small heat shock protein	1.706	1.063
clementine0.9_022028m/Ciclev10005897m	Embryo-specific protein	1.702	-0.777
clementine0.9_002926m/Ciclev10000352m	Glycosyl hydrolase family 3 protein	1.687	0.595
clementine0.9_008158m/Ciclev10028242m	Leucine-rich repeat family protein	1.673	0.380
clementine0.9_020929m	Peptidase m		0.173
clementine0.9_007997m/Ciclev10011525m	Aspartic proteinase	1.665	0.022
clementine0.9_015076m	Peroxidase 27		0.715
clementine0.9_005969m/Ciclev10025245m	LPR1 (Low Phosphate Root1)	1.613	-0.932
clementine0.9_012976m/Ciclev10008649m	Plastocyanin-like domain-containing protein	1.606	-0.018
clementine0.9_002976m/Ciclev10014355m	Subtilisin-like protease	1.603	0.329
clementine0.9_025727m/Ciclev10022906m	Inhibitor of trypsin and hageman factor	1.595	-2.133
clementine0.9_019843m/Ciclev10021884m	Expansin-like b1-like	1.583	-0.556
clementine0.9_024747m/Ciclev10029263m	Histone H2B	1.578	0.068
clementine0.9_020531m/Ciclev10002349m	Dehydrin 1	1.565	0.123
clementine0.9_008435m/Ciclev10015027m	Glycosyl hydrolase family 17 protein	1.544	-
clementine0.9_024798m/Ciclev10017092m	Eukaryotic translation initiation factor 1A	1.543	-
clementine0.9_026229m/Ciclev10017280m	NADH-ubiquinone oxidoreductase b18 subunit	1.525	-
clementine0.9_020331m/Ciclev10032615m	Auxin-induced in root cultures protein 12	1.524	1.011
clementine0.9_003766m/Ciclev10030843m	unknown protein	1.506	0.254
clementine0.9_008447m/Ciclev10015024m	SCPL40 (serine carboxypeptidase-like 40)	1.503	1.505
clementine0.9_025240m/Ciclev10006199m	60s ribosomal protein L22-2	1.502	0.336
clementine0.9_011579m/Ciclev10008467m	Eukaryotic translation initiation factor 4A1	-1.504	-
clementine0.9_022971m/Ciclev10026349m	Nicotinamidase	-1.508	-1.150
clementine0.9_023835m/Ciclev10032923m	ATP synthase d chain	-1.511	0.941
clementine0.9_001010m/Ciclev10007294m	Disease resistance protein (NBS-LRR class)	-1.515	-0.823
clementine0.9_011647m/Ciclev10001330m	DNAJ heat shock N-terminal domain-containing protein	-1.517	0.629
clementine0.9_001400m/Ciclev10018691m	Cell division cycle 5-like protein	-1.520	-0.375
clementine0.9_013575m/Ciclev10028695m	Pantothenate kinase	-1.527	-
clementine0.9_018717m/Ciclev10027333m	Calcium ion binding	-1.546	-0.299
clementine0.9_021984m/Ciclev10016269m	Syntaxin 23	-1.546	-0.001
clementine0.9_023333m/Ciclev10006024m	40s ribosomal protein s10-like	-1.567	0.336
clementine0.9_034011m	Benzoate carboxyl	-1.582	0.979
clementine0.9_029071m/Ciclev10006465m	FAD-binding domain-containing protein	-1.592	0.430
clementine0.9_012453m/Ciclev10025804m	Chalcone synthase 2	-1.603	1.088
clementine0.9_026642m/Ciclev10002973m	Acyl-CoA-binding protein 6	-1.613	0.642
clementine0.9_025382m/Ciclev10017167m	Programmed cell death protein 5	-1.629	0.359
clementine0.9_025950m/Ciclev10013051m	60s acidic ribosomal protein P2	-1.645	0.420
clementine0.9_001048m	Unknown protein	-1.669	-0.892
clementine0.9_021243m/Ciclev10005840m	Glutathione transferase	-1.672	-1.421
clementine0.9_007327m/Ciclev10024917m	Phospholipase D alpha 1	-1.686	-0.096
clementine0.9_002685m/Ciclev10019696m	Glutamate-cysteine ligase	-1.686	-0.129
clementine0.9_016514m/Ciclev10001924m	Meprin and TRAF homology domain-containing protein	-1.689	1.524
clementine0.9_003119m/Ciclev10014376m	Calcium-binding EF hand family protein	-1.724	-
clementine0.9_018424m/Ciclev10016241m	Proteasome subunit beta type	-1.751	0.191
clementine0.9_017608m/Ciclev10032291m	Alpha-soluble NSF attachment protein	-1.779	0.186
clementine0.9_028964m	Beta-caryophyllene synthase	-1.880	-0.796
clementine0.9_025957m/Ciclev10033122m	Tubulin-specific chaperone A	-1.890	0.057
clementine0.9_026048m/Ciclev10013168m	Vacuolar ATP synthase subunit G 1	-1.908	-
clementine0.9_014859m/Ciclev10008832m	Farnesyl pyrophosphate synthetase	-1.916	-
clementine0.9_014472m/Ciclev10028483m	Vacuolar sorting protein 4b	-1.923	0.745
clementine0.9_007126m/Ciclev10000432m	Unknown protein	-2.045	-0.348
clementine0.9_024367m/Ciclev10016953m	-	-2.288	-0.230
clementine0.9_020940m/Ciclev10029230m	Metal ion binding protein	-2.421	0.775

*: ‘/’ separates the IDs of the same gene (old version (Cclementina_165)/new version (Cclementina_182));-: not available

**Fig 1:**
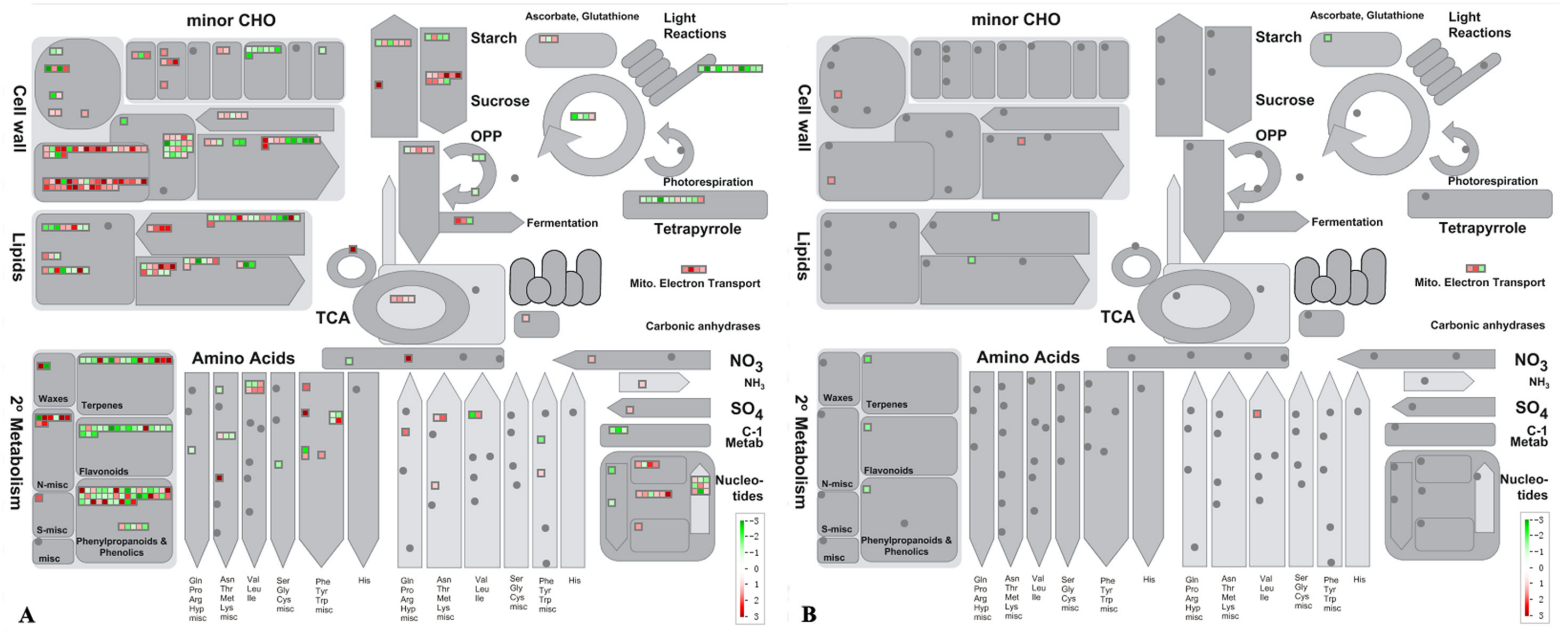
Mapman analysis for differentailly expressed genes (A) and diferentially expressed proteins (B) involved in metabolic pathways. Red squares represent genes or proteins that were significantly up-regulated; green squares represent genes or proteins that were significantly down-regulated.

Of the DEPs, a sperm specific protein 411 (SSP411) (Ciclev10018836m) was noteworthy for the protein was up-regulated by more than 6-fold while its mRNA level was slightly decreased by about 1.5-fold in HLB-infected roots. The copper ion binding protein (Ciclev10024168m) was increased by 4.78-fold, and a concomitant increase of 5.86-fold was also found for its transcripts. A germin-like protein (Ciclev10030149m) showed a 4.1-fold and a 4.78-fold increases in its protein and mRNA levels, respectively. Another germin-like protein (Ciclev10018003m) was similarly up-regulated (increased by 3.3-fold in protein level and 3.4-fold in mRNA level). A SCPL40 (serine carboxypeptidase-like 40) protein was up-regulated by > 3-fold and > 8-fold respectively in protein and mRNA levels. Two of the 4 subtilisin-like protease DEPs were found to be also up-regulated in their mRNA level but the other two were not. We identified also 3 up-regulated DEPs (leucine-rich repeat family protein, glycosyl hydrolase family 3 protein, expansin-like B1) in roots (Fig 1 and S2 Table). Interestingly, more than 10% of the DEPs were associated with protein degradation (Table 2 and S2 Table).

### Gene pathway enrichment analysis of *C*Las-modulated host pathways

PageMan analysis showed that no significantly enriched pathways were associated with the DEPs identified in this study. However, this was not the case when the same analysis was applied to DEGs since not only down-regulated pathways but also up-regulated pathways were identified (Table 3). The up-regulated pathways were cell wall modification, cysteine and aspartate protease mediated protein degradation, sugar and nutrient physiology signaling, receptor kinases signaling, and calcium signaling while the down-regulated pathways were regulation of transcription and ubiquitin-dependent protein degradation. In addition, 3 down-regulated protein gene families, *cytochrome P450*, *UDP glucosyl and glucoronyl transferases* (*UGTs*) and *pentatricopeptide repeat containing proteins* (*PPRRPs*), and one up-regulated *β-1*, *3-glucan hydrolase* gene family (*βGlu*) were classified into the ‘not assigned’ by PageMan.

**Table 3:** PageMan display of *C*Las-modulated pathways identified by RNA-seq.

Bin	Bin description	p-value
**30**	**Signaling**	**2.83E-05**
**10.7**	**Cell wall. modification**	**1.06E-04**
35.1.5	Not assigned. no ontology. PPRRP	1.40E-04
29.5.11	Protein. degradation. ubiquitin	1.51E-04
29.5.11.4	Protein. degradation. ubiquitin.E3	3.88E-04
**30.3**	**Signaling. calcium**	**3.88E-04**
26.1	Misc. cytochrome P450	5.27E-04
**30.2**	**Signaling. receptor kinases**	**6.99E-04**
**30.2.17**	**Signaling. receptor kinases. DUF 26**	**0.001199**
27.3	RNA. regulation of transcription	0.00317
29.5.11.4.3.2	Protein. degradation. ubiquitin. E3. SCF. FBOX	0.003335
29.5.11.4.3	Protein. degradation. ubiquitin. E3. SCF	0.00392
27	RNA	0.004422
**29.5.3**	**Protein. degradation. cysteine protease**	**0.009876**
**29.5.4**	**Protein. degradation. aspartate protease**	**0.009876**
**30.1**	**Signaling. in sugar and nutrient physiology**	**0.009935**
35.1	Not assigned. no ontology	0.019032
27.3.8	RNA. regulation of transcription. C2C2(Zn) DOF zinc finger family	0.034666
**26.4**	**Misc. beta-1,3-glucan hydrolases**	**0.038032**
26.2	Misc. UDP glucosyl and glucoronyl transferases	0.049359

Note: Up-regulated pathways are highlighted in bold; PPRRP: pentatricopeptide repeat containing protein; DUF: domain of unknown function; SCF: F-box containing complex; DOF: DNA binding with one finger.

**Fig 2:**
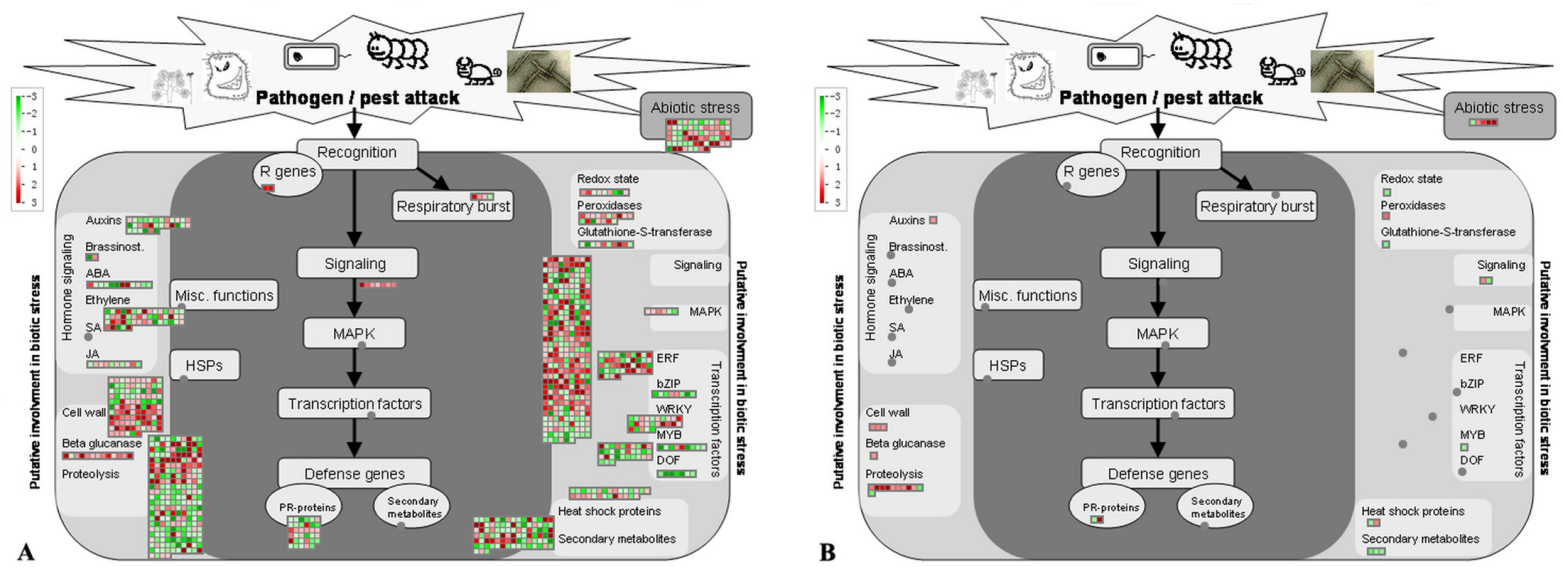
Mapman analysis for differentailly expressed genes (A) and diferentially expressed proteins (B) involved in stress response. Red squares represent genes or proteins that were significantly up-regulated; green squares represent genes or proteins that were significantly down-regulated.

Analysis showed the largest proportion of the DEGs were related to biotic and abiotic stress responses since 434 and 1164 DEGs were categorized into the ‘plant and pathogen interaction’ and the ‘stress-related’ when analyzed by KEGG (Table 1) and by MapMan (S1 Table and Fig 2), respectively. Representative DEGs were 2 *RIN4* (*RPM1 interacting protein 4*), 7 *MEKK1*, 5 *JAZ* (*Jasmonate-ZIM-domain protein*), 1 *HSP90* (*heat shock protein 90*), 3 *PR1* (*pathogenesis-related 1*), 1 *NPR1*, 1 *ALD1* (*AGD2-like defense response protein 1*), and 12 of the 13 *βGlu* that were the up-regulated. Notably, one of the *βGlu* genes, *BG1*, was even up-regulated by more than 10-fold. Some PR proteins such as PR3 and PR4 were also shown to be up-regulated.

Around 400 DEGs that were assigned to the category of ‘regulation of transcription’. Among them were 44 *AP2/EREBPs*, 42 *MYBs*, 30 *C2H2 zinc finger family proteins* and 28 *bHLHs*, which were either up- or down-regulated even if they were from the same family. However, DEGs from some families including 6 *ARF* and 6 *TCP* family members were all down-regulated, and those from the *C2H2 zinc finger family*, the *homeobox transcription factor family*, the *GRAS transcription factor family*, and the *bZIP transcription factor family* were mostly down-regulated. Contrastingly, DEGs from some other families including 1 *PUB23* and 1 *PUB24* of the *PHOR1* family were only up-regulated, and those from the *WRKY* domain transcription factor family were mostly up-regulated. In addition, many DEGs of this category were unclassifiable, including 2 *CDR1* (*constitutive disease resistance 1*) that were up-regulated by 5.2- and 4.6-fold respectively, and 1 *aspartyl protease family protein* (ciclev10020250m) that was up-regulated by 5.8-fold.

A large proportion of DEGs, totaled at 308, were related to cell signaling, including 113 *leucine rich repeat XI protein* (LRR) genes and 67 *DUF 26* (*domain of unknown function*) *receptor kinase* genes, and 39 calcium signaling genes. The up- and the down-regulated DEGs of LRR were more or less equal in number, but calcium signaling related DEGs and *DUF26s* were mostly up-regulated.

Two hundred and twenty four DEGs were assigned to the category of ‘protein degradation’ including 135 ubiquitin-related genes, 17 subtilase-related genes, 12 cysteine protease-related genes, 14 aspartate protease-related genes, 9 serine-protease-related genes and 16 AAA type ATPase-related genes. The ubiquitin-related DEGs were mostly down-regulated, but the protease-related DEGs were mostly up-regulated.

### Comparison between DEGs in roots and those in other tissues

A comparison was made between our results and those of leaves [16–22], fruits [23, 24] and stems and roots [9], and the results were summarized in Table 4. As shown in the table, DEGs from different tissues that have been analyzed so far were mostly involved in sugar and starch metabolism, cell wall metabolism, stress responses, hormone signaling, signaling and transcription factors, transport, cell organization and development, and protein metabolism.

**Table 4:** Comparison of *C*Las-regulated pathways in citrus leaves, fruits, stems and roots.

Pathways	Leaves	Fruits	Stems	Roots
**Sugar and starch metabolism**	**Up**: *AGPase*, *AAM*, *SS*, *GBSS* [22]; **Down**: *BAM*, *SPS* [20], *SUS* [21];	**Up**: *Invertase*, galactinol metabolism, **DR**: starch metabolism and raffinose synthesis [27]	**Up**: *APL3*, *GBSS*, *AMY*, *myo-inositol oxygenase*; **Down**: *BAM1*, *neutral invertase*, trehalose biosynthesis [9]	**Up**: *SS*, *AGPases*, *fructokinase*, *invertases*, *hexokinases*, trehalose biosynthesis and myo-inositol metabolism; **Down**: *PGSIP4*, *SUS3*, *HGL1*, *BAM3*, *BAM6*
**Cell wall metabolism**	Mostly up-regulated [22] or down-regulated [23]	Mostly up-regulated [27]	Mostly down-regulated [9]	**Up**: cell wall modification and pectin esterases related
**Stress responses**	Differentially regulated [20, 22]	Differentially regulated [27]	Mostly up-regulated [9]	Differentially regulated
**Hormone signaling**	Differentially regulated [22]	**Up**: ethylene, SA and JA related; **Down**: cytokinins and gibberellins related [27]	**Up**: SA and JA related; **Down**: ABA related [9]	**Down**: cytokinin, ABA and GA related; **DR**: JA, auxin and ethylene related
**Signaling and transcription factors**	Differentially regulated; **Up**: *MYB*s, *WRKYs*, *LRRs*; **Down**: *AP2* [17, 20]	Differentially regulated; **Up**: *leucine-rich repeat (LRR) receptor kinases*, *DUF26* and *WRKY*s [27]	Differentially regulated; **Up**: *receptor like kniases*; **Down**: calcium signaling [9]	**Up**: MYB [9], *DUF26*, *WRKYs*, calcium signaling and sugar and nutrient physiology signaling related; **Down**: light signaling; **DR**: *LRRs*
**Transport**	**Up**: *zinc transporters*; **Down**: major intrinsic proteins [17]	Differentially-regulated; **Up**: Sugar, sulfur, ABC transporter and ammonium related transporters [27]	**Up**: *metal*, *peptide*, *oligopeptides*, *ions phosphate and nitrate transporter*; **Down**: major intrinsic proteins [9]	**Up**: *Zinc*, *copper*, *some phosphate and metabolite transporters*; **Down**: *nitrate transporters*, major intrinsic proteins, sugar, sulfur, amino acids, nucleotide, metal, peptide and oligopeptides transport related
**Protein metabolism**	Down-regulated [17]	**Up**: Protein degradation and misfolding;**Down**: Protein synthesis [27]	Differentially regulated [9]	**Up**: Protein synthesis, protease mediated protein degradation, **Down**: protein degradation [9]; ubiquitin-dependent protein degradation
**Photosynthesis**	Down-regulated [17, 20, 25]	Up-regulated [26, 27]	Mostly down-regulated [9]	Mostly down-regulated
**Secondary metabolism**	Mostly up-regulated [25]	Differentially regulated [27]; **Down**: flavonoid biosynthesis [26]	Mostly up-regulated [9]	**Up**: alkaloid metabolism related; **Down**: Phenlypropanoids, isoprenoids and flavonoids metabolisms,
**Energy metabolism**	Up-regulated [20, 21]	Up-regulated [27]	Differentially regulated [9]	Up-regulated
**Cell organization**	Differentially regulated [20, 21]	Differentially regulated [27]	Differentially regulated [9]	Mostly up-regulated
**Cell cycle and cell division**	Down-regulated [22]	Down-regulated [27]	-	Down-regulated
**Phloem proteins**	**Up:** *PP2-B15* [20–22, 24]; *PP2-B10*, *PP2-B14* [24]	-	-	**Up:** *PP2-B10*, *2-B15*, *2-B11*, *PP2-A13*, *PP2-A1* and *PP2-A9*

Note: Up: up-regulated genes or pathways; Down: down-regulated genes or pathways; DR: pathways or genes differently regulated; the italic are genes;-: not available; AGPase: ADP-glucose pyrophosphorylase; AAM: alpha-amylase; BAM: Beta-amylase; SS: starch synthase; GBSS: granule bound starch synthase; SUS: sucrose synthase; SPS: sucrose-phosphate synthase; PGSIP4: plant glycogenin-like starch initiation protein; HGL1: heteroglycan glucosidase 1; APL3: ADP-glucose pyrophosphorylase large subunit 3; LRR: leucine-rich repeat; DUF: domain of unknown function; PP: phloem protein

The trends in expression of the DEGs identified in roots by this study were generally consistent with those identified in the above ground tissues. For example, most DEGs of sugar and starch metabolism related protein modification related proteins, WRKYs, and energy metabolism related proteins were commonly up-regulated, while those related to cell cycle and cell division down-regulated, in all tissues (Table 4, Figs 1 and 2). The *β-amylase* (*BAM*) and *sucrose synthase* (*SUS*) genes down-regulated in the above tissues were also down-regulated in roots. *Glucan synthase-Like 7* (*GSL7*), a gene tightly coexpressed with two *SUS* genes [29], was down-regulated not only in *C*Lam-infected leaves but also in our *C*Las-infected roots. It was reported that several *phloem protein 2* (*PP2*) genes were up-regulated in HLB-infected leaves [17–19, 21], and in this study, 6 *PP2* DEGs were up-regulated with *PP2-B10* and *PP2-B15* as the most highly up-regulated as shown by a up-regulation of 10- and 12-fold respectively in their transcript levels in *C*Las-infected roots (Table 4 and S1 Table). Most protein degradation related DEGs that were up-regulated in many HLB infected tissues were also up-regulated in roots, including those of *subtilases*, *cysteine proteases*, *aspartate proteases*, *serine proteases*, and *AAA-type ATPase family proteins*, and comparatively, more DEGs of these categories were present in roots than in other tissues. DEGs involved in hormone metabolism and signaling pathways were also similarly modulated by *C*Las in different tissues (Table 4).

**Fig 3:**
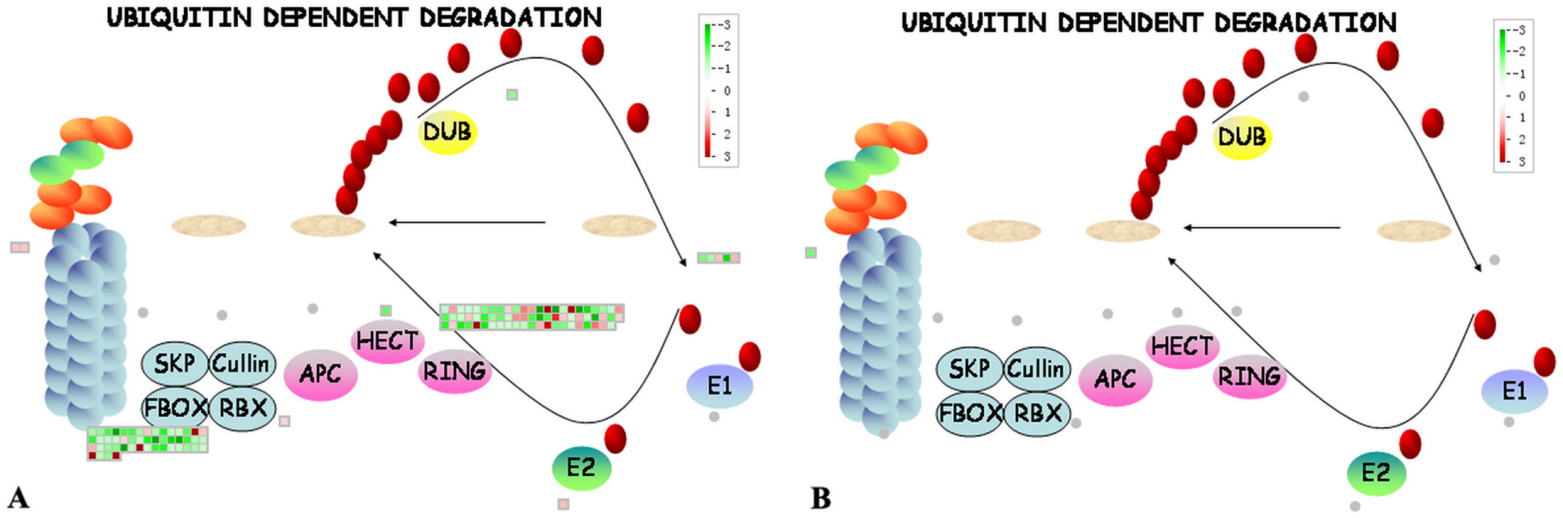
Mapman analysis for differentailly expressed genes (A) and diferentially expressed proteins (B) involved in ubiquitin-dependent protein degradation. Red squares represent genes or proteins that were significantly up-regulated; green squares represent genes or proteins that were significantly down-regulated.

**Fig 4:**
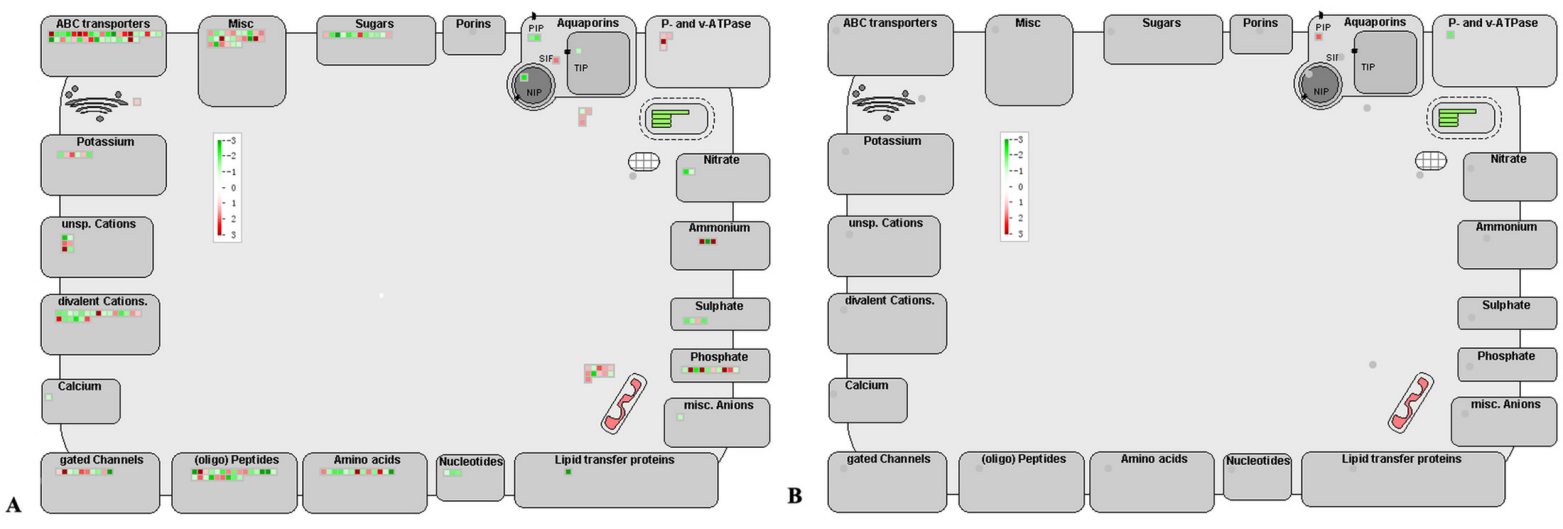
Mapman analysis for differentailly expressed genes (A) and diferentially expressed proteins (B) involved in transportation. Red squares represent genes or proteins that were significantly up-regulated; green squares represent genes or proteins that were significantly down-regulated.

However, some DEGs were oppositely regulated in roots as compared with one or more above-ground tissues. Some cell wall metabolism-related DEGs such as *pectin esterase* genes were down-regulated in stem [9] but most of them were up-regulated in leaves [19], fruits [24] and roots (Table 4). Photosynthesis related genes down-regulated in leaves and stems were also down-regulated in non-photosynthetic roots as shown in this study (Table 4) but they were up-regulated in fruits [23, 24]. On the contrary, calcium signaling related DEGs were down-regulated in stems [9] but most of them were up-regulated in roots (Table 4). Ubiquitin-dependent protein degradation related genes were either not regulated or up-regulated in the above ground tissues [24] but most of them were down-regulated in roots (Fig 3). Similarly, most of the secondary metabolism related genes were up-regulated in the above ground tissues [9, 22], but they were mostly down-regulated in roots. Nearly 3/4 of the 104 *cytochrome P450 monooxygenase* DEGs were down-regulated in roots (S1 Table). But one of them, *CYP83B1*, whose protein is involved in biosynthesis of glucosinolates and callose deposition [30] was up-regulated in *C*Lam-infected leaves [21]. Although some of the 7 *CYP83B1* DEGs were also up-regulated in HLB-infected roots, the remaining others were down-regulated. A total of 203 transport related DEGs were identified in roots (Fig 4), but comparatively there were only 39 of them in stems [9]. Moreover, far more HLB-regulated ABC transporter DEGs were found in our study. Z*inc transporter precursor* (*ZIP*) genes were up-regulated in leaves [21], and agreeably, 3 up-regulated *ZIP* genes were identified in roots. Five of the 10 phosphate transport-related DEGs including 2 *PHT3* (*phosphate transporter 3*), 1 *PHT1;1*, 1 *PHT4;6*, and 1 *phosphate transmembrane transporter* were up-regulated, and *PHT3* and the *PHT1* were even up-regulated by more than 7-fold but the rest 5 were down-regulated in roots; in comparison, only one of them, *PHT3*, was found to be up-regulated in stems [9]. Two nitrate transporter genes, *NRT2*:*1* (*nitrate transporter 2*:*1*) and *NRT2*:*5*, were both down-regulated in roots whereas two different nitrate transporter genes, *NRT1* and *NRT1*:*2*, were up-regulated in stems [9]. We identified also 2 highly up-regulated *AMT2* (*ammonium transporter 2*) genes (ciclev10023275m, 4.6-fold; ciclev10010379m, 12-fold) and one highly down-regulated *AMT1*:*2* (ciclev10019808m, -4-fold) while previous reports did not mention any of them as DEG.

Root specific changes were also observed for many DEGs. For example, many metal transporter genes were down-regulated while only a few or no such DEGs were identified in the above ground tissues. Three *ferric reduction oxidase* (*FRO*) genes, *FRO2*, *FRO7* and *FRO8*, were down-regulated in roots but not significantly changed in the above ground tissues of the HLB-infected trees. Another striking finding in our root results was that as many as 94 DEGs, 86 of which were down-regulated, were from the largest plant protein gene family *PPRRP* since no such DEGs were reported in previous studies involving the above ground tissues.

### Changes in expression of DEGs in roots following *C*Las infection

**Fig 5:**
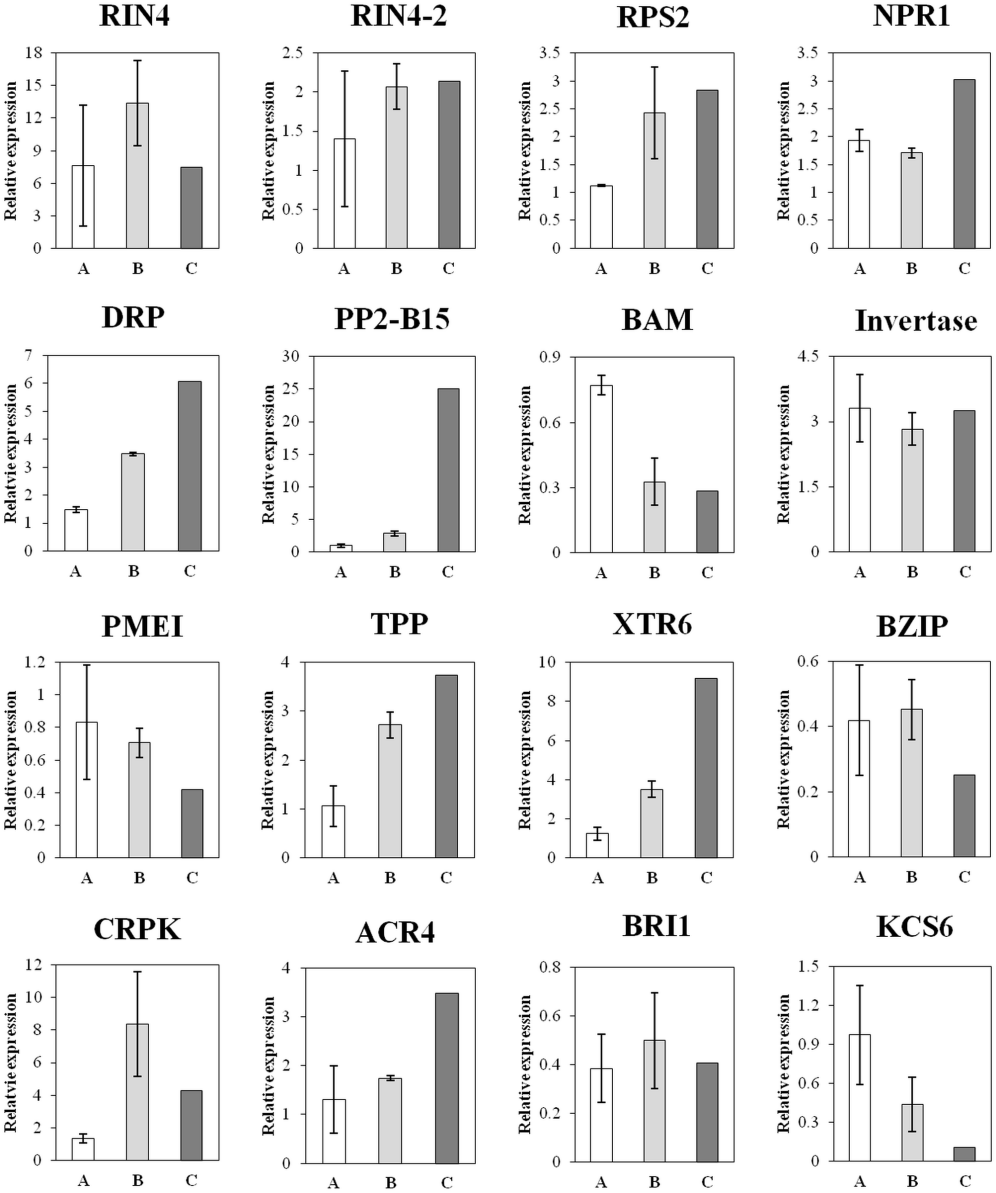
Expression of 16 differentially expressed genes at 20 dpi (A) and 50 dpi (B) as determined by quantitative real time PCR. C indicates the expression level determined by RNA-seq. *RIN4*: *RPM1 interacting protein 4*; *RPS2*: *disease resistant protein ribosomal protein S2*; *NPR1*: *regulatory protein nonexpresser of PR genes 1*; *DRP*: *disease resistant protein (TIR-NBS-LRR class)*; *PP2-B15*: *Phloem protein 2-B15*; *BAM*: *β-amylase*; *PMEI*: *pectin methylesterase inhibitor*; *TPP*: *trehalose-6-phosphate phosphatase*; *XTR6*: *Xyloglucan endotransglycosylase 6*; *BZIP*: *bZIP transcription factor*; *CRPK*: *cysteine-rich protein kinase*; *ACR4*: *Act repeat 4*; *BRI1*: *BRI1 kinase inhibitor 1*; *KCS6*: *3-ketoacyl-CoA synthase 6*.

Quantitative real-time PCR (qRT-PCR) was performed to investigate the changes in transcription of 16 typical HLB-modulated genes in roots at 20 dpi and 50 dpi. As shown in Fig 5, the expression of all the investigated genes in roots of 50 dpi was consistent between qRT-PCR and RNA-seq data, showing that the quality of our RNA-seq data was acceptable. Comparison of the data between the two time points revealed 4 expression patterns: 1) a almost constant up-regulation at both 20 dpi and 50 dpi, as represented by *NPR1*, *invertase* and *ACR4*; 2) a moderate increase at 20 dpi followed by a more significant increase at 50 dpi as represented by *RIN4*, *RPS2*, *DPI*, *PP2-B15*, *TPP*, *XTR6*, *CRPK*; 3) a moderate down-regulation at 20 dpi followed by a more significant down-regulation at 50 dpi as represented by *BAM*, *KCS6* and *PMEI*; and 4) a initial significant down-regulation at 20 dpi followed by a moderate down-regulation at 50 dpi as represented by *BRI1* and *BZIP*.

## Discussion

HLB bacteria can be vectored from diseased trees to healthy trees by ACP (Asian citrus psyllids), a species of tiny insect that is difficult to control in open field [31]. Therefore, the hope of bringing the disease under control relies on developing new cultivars with HLB resistance [26]. However, facing with the problems of long juvenile period, large tree size, frequently occurred polyembryonic seeds, and no available resistance genes, breeding for HLB resistant citrus by hybridization has so far made little progress. Alternatively, transgenic citrus should intimately offer a solution to the problem [32]. But efficient transgenic engineering work requires knowing where the host innate defense system is breached by the invading bacteria. Studies have showed that changes in transcriptome and proteome in host following pathogen infection can provide important clues to the mechanism of pathogenesis and suggest candidate genes or pathways for engineering for resistance. In *Citrus*, transcriptomic studies have been conducted in leaves, stems and fruits of HLB-infected trees [9, 16–24], and proteomic study was also conducted in leaves [16]. These studies have accumulated a large body of information about the molecular mechanism of HLB-host interactions. However, roots are largely neglected though evidence showed that roots are equally susceptible to and can be quickly colonized by HLB bacteria [3, 27]. The only transcriptional analysis involving roots of HLB-infected Valencia orange showed that only 111 DEGs were detected by microarray [9]. Given the importance of roots in absorption and transportation of nutrients and in offering mechanical support to the whole tree, roots may differ significantly in their responses to HLB-infection than other tissues.

In this study we used respectively RNA-seq and iTRAQ to investigate the changes in transcriptome and proteome of ‘Sanhu’ red tangerine roots following inoculation with HLB bacteria. Our results showed that HLB-infection changed significantly the expression of 3956 genes in roots, a number that is much larger than that obtained by microarray analysis [9]. The vast discrepancy in DEG numbers should be related to the different sensitivities of the two methods: microarray is less sensitive since the hybridization signals of certain probes can be either saturated by over representative genes or too weak to be detected for rare transcripts [33]; in contrast, RNA-seq counts the absolute number of the transcripts and therefore the sensitivity can always be guaranteed by increasing sequencing coverage [34].

We also detected 78 differentially regulated proteins in this study. A moderate positive correlation was found between our DEP and DEG data, indicating HLB-induced changes in gene expression are regulated at both transcriptional and post-transcriptional levels. Given that our sampling date of 50 dpi was earlier than other previous transcriptomic researches [9, 16–24] and that it takes time for cells to mature mRNAs and to translate them, it may also be possible that the genes’ expression changes may have not yet led to a significant turnover in their protein levels at 50 dpi.

Comparison between our data and those obtain in other studies [9, 16–24] reveled that many common genes and hence the relevant pathways are regulated in both the above ground and the underground tissues (Table 4). These pathways included the stress-response pathways that are up-regulated, the cell-wall modification pathways that are up-regulated, the photosynthesis pathway that is repressed except in fruits, the protease-mediated protein degradation pathway that is down-regulated, the cell cycle and cell division related genes that are mostly down-regulated, the zinc transporters that are up-regulated, the phloem proteins that are up-regulated in leaves and roots, among others. However, some responses of root-specific or opposite to those of the above ground tissues did exist as discussed in the following sections.

### HLB-infection reduced the overall defense capability in roots of the host

The most eye-catching changes were the down-regulation in terpenoid, flavonoid and phenylpropanoid biosynthesis pathways in roots following HLB infection since many of the pathways’ biosynthesis genes were down-regulated. Flavonoids are anti-fungi substances and antioxidants [35, 36]. Phenylpropanoids serve as structural polymers including lignins, provide protection from pests and UV light and attract pollinators as pigments [37]. Important terpenoids include GA and ABA, carotenoids and some anti-pest chemicals [38]. Taken together, the defense of the root cells may be greatly weakened from a reduced biosynthesis of defensive substances in the host by HLB bacteria at the very early stage of their infection.

Plants use a diverse array of cytochrome P450 monooxygenases in biosynthesis of secondary metabolites and in detoxification [39]. It was shown in this study that most of the proteins’ genes were down-regulated, which may signal a reduced biosynthesis of secondary metabolites and a reduced reducing power in HLB-infected roots.

A notable finding in this study was that the negative regulator of plant defense against bacterial infection, *RIN4* [40], was up-regulated in HLB-infected roots at both 20 dpi and 50 dpi. RIN4 was shown to be associated with two NB-LRR immune receptors, RPS2 and RPM1, in planta [41, 42]. It can be cleaved by avrRpt2 to activate RPS2-dependent resistance [42]. It can also be phosphorylated indirectly by avrB and avrRpm1 to activate RPM1 which in turn activates resistance responses [40]. The gene’s up-regulation indicated that HLB bacteria may harness the gene to facilitate their colonization.

PPRRPs constitute the largest protein super family in planta and are mostly located in organelles such as mitochondria and chloroplast [43–45]. The down-regulation in expression of a very large proportion of *PPRRP*s may indicate that a shortage in energy supply occurred in *C*Las-infected roots since PPRRPs are generally involved in proton-electron translocation in mitochondria. It may also indicate a decreased oxidative respiration that otherwise enhances stress tolerance [45, 46].

### HLB-infection reduced greatly the absorption of N, P, Zn and Fe

As roots are the sole organ to absorb and supply inorganic nutrients for the whole tree, the nutrient deficiencies in leaves should be closely related to the function of roots. It was reported that zinc and phosphorus content was significantly reduced in HLB infected trees [14, 25] and that Zn deficiency induced the expression of genes encoding phosphate transporters in barley roots [47]. Our study showed that zinc and phosphate transporter genes were similarly up-regulated in roots as in leaves and stems [9, 21]. Whether this up-regulation is resulted from zinc or/and phosphorus deficiency requires further investigation. Two nitrate transporter genes, *NRT1* and *NRT1*:*2* were reported to be up-regulated in stems [9] but our result showed *NRT2*:*1* and *NRT2*:*5* were down-regulated in roots. Apparently, HLB infected trees are deficient in nitrogen, which accounts largely for the characteristic chlorosis symptom [15]. And a down-regulation in nitrogen transporter genes in roots strongly indicated a reduced supply of the element to the above ground tissues. Fe is essential for photosynthesis and chlorophyll synthesis, and deficiency in Fe results in leaf chlorosis. Fe as cofactor is also necessary for some enzymes to function normally. It was reported that Fe in some HLB-infected citrus genotypes was only half of the level of the healthy plants, indicating that Fe homeostasis is significantly affected during infection [14]. In our study 3 genes encoding FRO2, FRO7 and FRO8 were all down-regulated by *C*Las-infection, strongly suggesting that the normal Fe absorption and transportation are impaired in roots for these ferric chelate reductases are responsible for reducing insoluble Fe^3+^ to soluble Fe^2+^ [48]. Clearly, our data can explain, at least in part, for the leaf chlorosis symptom associated with HLB infection.

### HLB-infection can nevertheless induce some defense responses

Phloem protein 2 (PP2) participates in the formation of high-molecular-weight polymers that plug the sieve plate pores of injured sieve tube elements [49, 50], and shows also *in vitro* [51] as well as *in vivo* [52] insecticidal activities. *PP2-like protein* genes were reported in HLB-infected leaves [16–21]. We also identified a strong up-regulation in the expression of 6 *PP2-like* genes in roots. Our qRT-PCR result confirmed that the *PP2-B15* gene was induced quickly at 20 dpi and more significantly at 50 dpi. It therefore seemed that the increased expression in many *PP2-like* genes following *C*Las infection has an active role in defense against the invading bacteria and perhaps even the feeding psyllids. Previous studies indeed proposed that these proteins were responsible for the widely observed callose deposition in the infected sieve tube elements in leaves [9, 18]. However, *GSL7*, a callose synthase gene that is necessary for normal phloem carbohydrate transport and inflorescence growth [29], was down-regulated in HLB-infected roots as shown in this study (S1 Table), possibly signifying a reduced transport of assimilates in the infected phloem tube elements, which might be result in carbohydrate starvation as shown in the *Arabidopsis* mutant *gsl7*. Interestingly, the expression of *βGlu*, whose products hydrolyze callose [53], was up-regulated by *C*Las-infection (Table 2), indicating again a disturbed callose lining of the sieve plate pores that is required for normal carbohydrate transportation. The contradictory regulation in callose synthesis and degradation genes may indicate that cells are hanging between victory and defeat in their fight against *C*las at this stage.

Of the dozens of *UGT* DEGs that were significantly regulated in *C*Las-infected roots, more were down-regulated than up-regulated (S1 Table). But the few *UGT*s detected in HLB-infected above ground tissues were mostly up-regulated [18, 21, 24]. The exact role of these UGTs in response of citrus to *C*Las-infection is not clear since knocking out certain *UGTs* enhanced defense against *Pseudomonas syringae* but knocking out some other *UGTs* increased susceptibility to the same bacterium in *Arabidopsis* [54, 55].

SA signaling pathway may be activated in *C*Las-infected roots since the key SA signaling-related gene *NPR1* (ciclev10031627m) was up-regulated and one of the pathway’s downstream genes [56], *PR1*, was concomitantly up-regulated. Further more, 2 PR proteins, PR3 and PR4, were also up-regulated. *ALD1*, a gene that was reported to be activated by SA, was up-regulated [57]. In addition, several *WRKY transcription factor* genes that participate in SA- and JA- dependent defense pathways were up-regulated [58].

Proteolysis is fundamental for the normal functioning of multicellular organisms and plays key roles in a variety of biological processes including defense and stress responses [59]. Recently, the possible role of subtilisin-like protease, a serine protease, in plant-pathogen recognition and immune priming has been suggested [60]. CDR1, a secreted aspartic protease, was also reported to function in plant defense responses [61]. Aspartic and cysteine proteases were demonstrated to be associated with defense [62, 63]. Therefore it was not surprising to find that the genes of these proteases were regulated in HLB-infected roots (S1 Table and Table 2). The surprise was, however, that some of their corresponding proteins were elevated to a level that was high enough to allow them to be identified as of the mere 78 DEPs. But the ubiquitin-dependent protein degradation pathway, as discussed in above, was down-regulated in HLB-infected roots. In this respect, it was known that some pathogens have acquired during evolution the ability to subvert the host’s proteasomal degradation pathway to facilitate their infection [64], suggesting that *C*Las may have also evolved the same strategy to counteract the attacks of its hosts.

Several other genes (and their encoded proteins) that were highly up-regulated in *C*Las-infected ‘Sanhu’ red tangerine roots (Table 2) included several *germin-like proteins* and 1 *SCPL40*. Germins and germin-like proteins could be induced in the resistance response of plants to bacterial infections [65]. SCPLs have recently emerged as a new group of plant acyltransferases that are required for the synthesis of antimicrobial compounds [66, 67]. The facts that the highest and the second highest up-regulated proteins were SSP411 and the copper ion binding protein in HLB-infected roots (Table 2) strongly suggested that the proteins play some important roles in the response of citrus to *C*Las-infection.

## Materials and Methods

### Plant materials and treatments

Two-year-old seedlings of ‘Sanhu’ red tangerine were grafted with buds from *C*Las-infected or *C*Las-free ‘Gonggan’ mandarin trees. Mature leaves and roots were collected from the *C*Las-inoculated and the control trees every ten days to detect for *C*Las by PCR. DNA used for PCR was extracted with the use of the Plant DNA isolation Kit (Trans, Beijing, China) according to manufacturer’s instructions. Primers used for *C*Las detection were the same A2/J5 as reported by Hocquellet *et al*. [68].

### RNA-sequencing and iTRAQ analysis

The fibrous roots of 3 *C*Las-positive and 3 control *C*Las-free trees were individually collected at 50 dpi when the HLB-inoculated trees became *C*Las-positive in both leaves and roots yet showed no visible chlorosis and other HLB symptoms. This should have allowed us not to miss too many early responsive genes but at the same time ensured that the trees were infected as expected. Total RNA was extracted from each sample using RNeasy plant mini kit (Qiagen, Valencia, CA) and further purified using the RQ1 Rnase Free Dnase Kit (Promega, Madison, USA). RNA quality and quantity were assessed by gel-electrophoresis and by absorbance at OD260/OD280, respectively. Aliquot RNA samples were stored at -80°C. For RNA-seq analysis, RNA samples from the three trees were mixed in equal amount and used for cDNA library construction following the Illumina mRNA-sequencing sample preparation protocol (Illumina, San Diego, CA). The 90-bp raw paired end reads were generated by Illumina HiSeq 2000. The RNA-Seq data have been submitted to the Gene Expression Omnibus (GEO) database (http://www.ncbi.nlm.nih.gov/geo/info/linking.html.); Accession number GSE67560.

Total proteins were also extracted from the same samples and subject to iTRAQ labeling, SCX chromatography fractionation and LC-ESI-MS/MS analysis with the same method as described by Yang *et al*. [69].

### Data analysis

RNA-sequencing data were filtered to remove low quality reads. The clean individual reads from both the *C*Las infected and the control libraries were aligned first to the *C*. *clementina* genome and then to all transcript sequences (http://www.phytozome.org.) in 2011 with a mismatch penalty of no more than 1 nucleotide. For quantitative gene expression analysis, the transcripts of each gene were normalized to RMPK (reads per kb per million reads) and the DEGs were further annotated using Blast2GO and MapMan [70, 71], and the MapMan results were shown in S1 Table.

Raw iTRAQ data files acquired from the Orbitrap were converted into MGF files using Proteome Discoverer 1.2 (PD 1.2, Thermo). Subsequent database searches were carried out by Mascot Daemon (version 2.3.02, Matrix Science, Boston, MA) for both protein identification and iTRAQ quantification against the *C*. *clementina* (Cclementina_165). The quantitative protein ratios were weighted and normalized by the median ratio with outlier removal in Mascot in which the imbedded isotope correction factors were applied. Proteins with a ≥ ∣±1.5∣-fold change (*C*Las-infected vs. control) were defined as differentially expressed proteins (DEPs). The functional annotation and classification of all DEPs were performed by using Blast2GO and MapMan and the MapMan results were summarized in S2 Table.

To identify and to provide a statistics-based overview of the changed pathways, the DEGs and DEPs were analyzed by PageMan embedded in MapMan. The Wilcoxon test was applied and the Benjamini and Hochberg approach corrected p-value (<0.05) were generated [72]. Pearson method was used to analyze the correlation between the levels of DEPs and those of their corresponding mRNAs.

### Quantitative real time PCR

Reverse transcription was performed using total RNA from each biological replicates as template using the PrimeScript RT reagent Kit with gDNA Eraser (Perfect Real Time) (Takara, Dalian, China) according to the manufacturer’s instruction. The 16 genes selected include 11 up-regulated genes (two *RPM1 interacting protein 4* (*RIN4*), *invertase*, *Phloem protein 2-B15* (*PP2-B15*), *NPR1 regulatory protein (NPR1)*, *trehalose-phosphate phosphatase-like (TPP)*, *act repeat 4* (ACR4), *disease resistant protein ribosomal protein S2* (*RPS2*), *disease resistance protein TIR-NBS-LRR class (DPI)*, *cysteine-rich protein kinase* (*CRPK*), *xyloglucan endotransglucosylase hydrolase* (*XTR6*)) and 5 down-regulated genes (*bri1 kinase inhibitor 1* (BRI1), *bzip transcription protein factor-like* (BZIP), *3-ketoacyl-CoA synthase 6* (*KCS6*), *β-amylase* (BAM) and *pectin methylesterase inhibitor* (*PMEI*)) identified by RNA-seq. qRT-PCR was carried out in Lightcycler 480II (Roche, Switzerland) using SYBR Green real time PCR Master Mix (Toyobo, Osaka, Japan) according to Cheng *et al*. [73]. *Actin* gene was used as the endogenous control. The expression was calculated by 2^-ΔΔCt^ and normalized against *actin* gene expression level. Genes analyzed and their primers used were listed in S3 Table.

## Supporting Information

S1 Fig Pearson correlation analysis result between DEGs and DEPs in HLB infected roots. (TIF)

S1 Table Mapman analysis results of the differentially expressed genes in HLB-infected roots in comparison with control roots. (XLS)

S2 Table Mapman analysis results of the differentially expressed proteins in HLB-infected roots in comparison with control roots. (XLS)

S3 Table Primers for real time PCR analysis. (DOCX)
