# Supplementary figures and images for: Comparative Transcriptome and iTRAQ Proteome Analyses of Citrus Root Responses to Candidatus Liberibacter asiaticus Infection
Source: PLoS One. 2015 Jun 5;10(6):e0126973. doi: 10.1371/journal.pone.0126973 (PMC4457719; doi:10.1371/journal.pone.0126973)

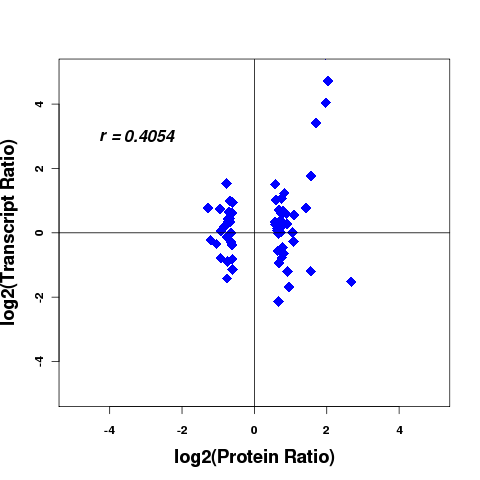

Supplement: S1 Fig — (TIF) [file pone.0126973.s001.tif]
