# Supplementary material for: Comparative Transcriptome and iTRAQ Proteome Analyses of Citrus Root Responses to Candidatus Liberibacter asiaticus Infection
Source: PLoS One. 2015 Jun 5;10(6):e0126973. doi: 10.1371/journal.pone.0126973 (PMC4457719; doi:10.1371/journal.pone.0126973)
Supplement: S3 Table — (DOCX) [file pone.0126973.s004.docx]

**S3 Table. Primers for real time PCR analysis**

| **Gene ID** | **Gene description** | **Forward Primer** | **Reverse Primer** |
| --- | --- | --- | --- |
| **Ciclev10027348m** | RIN4 | F: TGTCATCTCATGGTCACGGAAGCAC | R: ACCCCTTTTCTCTTCACTGGCCTTG |
| **Ciclev10022221m** | RIN4-2 | F:AAGAGGGTAGTGATCTCAGGCAGTC | R: TCACCAGAACTTGCTACACGACCTC |
| **Ciclev10031627m** | NPR1 nim1-like regulatory protein | F: GCAAGGCGTAATGAGCCAGAAGTGA | R: TTAGTTCCCTGCCCCTGCTTTGTAG |
| **Ciclev10001513m** | trehalose-phosphate phosphatase-like | F: TCCGTACACTTCCGTTGCGTTGATG | R: CTTACTTGCTCGGCTAATGTACCCC |
| **Ciclev10031536m** | act repeat 4 | F: TGACTGATGAAGAAACTGGGGGTGC | R: TTTGGTGAAGCCTCCTCTCAGTGTG |
| **Ciclev10027214m** | RPS2 | F:GTGGAGTGTTTGGGAGACAACGAAG | R: GGAATATCAGGATGACTGCCGAGAG |
| **Ciclev10023454m** | Disease resistant protein (tir-nbs-lrr) | F: AAAGGCTCCAACGGACAAAGGTGCT | R: TACCCAGACCAAATCGTTCAAGCCC |
| **Ciclev10014521m** | cysteine-rich receptor like protein kinase | F: CGGCAGAGGTTGTTGGAAAGGCTAT | R: TGCAATCAGCTTCGGATAGGTCAGG |
| **Ciclev10010858m** | xyloglucan hydrolase endotransglucosylase | F: CATCTTGCGCTTCCTCTAAGTCCAG | R: TGGGGAAATCGCTTTGTGTCAGTGC |
| **Ciclev10003734m** | Invertase | F:GCTATTTTATCGGCAATGTGAGTCC | R:CCATCGTGCTTCAATAAGGTCCATA |
| **Ciclev10013351m** | PP2-B15 | F: GATTAACATCACCCACCGTGCACAT | R: CGGTTGCCGTAAAACAAACACTCCA |
| **Ciclev10001740m** | bri1 kinase inhibitor 1 | F: GGGTTCACAAGATGGCGAAAGGGTT | R: ACGAATACGCCTGCCGTTGGAAATG |
| **Ciclev10031361m** | bzip transcription factor | F: CATGGTGGCCAAGACGGATGTGTTT | R:GGAATCCACCCAACCACAAGAAGCA |
| **Ciclev10031329m** | 3-ketoacyl- synthase | F: CGGAGCAGTTGCTGTTTCTCATGAC | R: ACTCATCAATCACCGCTCTTCCACC |
| **Ciclev10004689m** | beta-amylase | F: GGATAATGGTGGTTCCTGGGAGTCT | R:CGCCAAAAGTTGATGAGGCAAGGGA |
| **Ciclev10023652m** | invertase inhibitor-like | F: GGTGCCTACTCTGAAACAAGCCATG | R: CTTCACAAGTGGTGCAGGCATCAAC |
| Internal Reference | Actin | F: CCAAGCAGCATGAAGATCAA | R: ATCTGCTGGAAGGTGCTGA |
